# A dynamics association study of gut barrier and microbiota in hyperuricemia

**DOI:** 10.3389/fmicb.2023.1287468

**Published:** 2023-11-27

**Authors:** Qiulan Lv, Jun Zhou, Changyao Wang, Xiaomin Yang, Yafei Han, Quan Zhou, Ruyong Yao, Aihua Sui

**Affiliations:** ^1^Medical Research Center, The Affiliated Hospital of Qingdao University, Qingdao, China; ^2^Laboratory Medicine, The Affiliated Hospital of Qingdao University, Qingdao, China

**Keywords:** hyperuricemia, gut microbiota, intestinal barrier, dynamic changes, dyslipidemia

## Abstract

**Introduction:**

The intricate interplay between gut microbiota and hyperuricemia remains a subject of growing interest. However, existing studies only provided snapshots of the gut microbiome at single time points, the temporal dynamics of gut microbiota alterations during hyperuricemia progression and the intricate interplay between the gut barrier and microbiota remain underexplored. Our investigation revealed compelling insights into the dynamic changes in both gut microbiota and intestinal barrier function throughout the course of hyperuricemia.

**Methods:**

The hyperuricemia mice (HY) were given intragastric administration of adenine and potassium oxalate. Gut microbiota was analyzed by 16S rRNA sequencing at 3, 7, 14, and 21 days after the start of the modeling process. Intestinal permeability as well as LPS, TNF-α, and IL-1β levels were measured at 3, 7, 14, and 21 days.

**Results:**

We discovered that shifts in microbial community composition occur prior to the onset of hyperuricemia, key bacterial *Bacteroidaceae*, *Bacteroides*, and *Blautia* exhibited reduced levels, potentially fueling microbial dysbiosis as the disease progresses. During the course of hyperuricemia, the dynamic fluctuations in both uric acid levels and intestinal barrier function was accompanied with the depletion of key beneficial bacteria, including *Prevotellaceae*, *Muribaculum*, *Parabacteroides*, *Akkermansia*, and *Bacteroides*, and coincided with an increase in pathogenic bacteria such as *Oscillibacter* and *Ruminiclostridium*. This microbial community shift likely contributed to elevated lipopolysaccharide (LPS) and pro-inflammatory cytokine levels, ultimately promoting metabolic inflammation. The decline of *Burkholderiaceae* and *Parasutterella* was inversely related to uric acid levels, Conversely, key families *Ruminococcaceae*, *Family_XIII*, genera *Anaeroplasma* exhibited positive correlations with uric acid levels. *Akkermansiaceae* and *Bacteroidaceae* demonstrating negative correlations, while LPS-containing microbiota such as *Desulfovibrio* and *Enterorhabdus* exhibited positive correlations with intestinal permeability.

**Conclusion:**

In summary, this study offers a dynamic perspective on the complex interplay between gut microbiota, uric acid levels, and intestinal barrier function during hyperuricemia progression. Our study suggested that Ruminiclostridium, Bacteroides, Akkermansiaceae, Bilophila, Burkholderiaceae and Parasutterella were the key bacteria that play vital rols in the progress of hyperuricemia and compromised intestinal barrier, which provide a potential avenue for therapeutic interventions in hyperuricemia.

## 1 Introduction

Hyperuricemia, clinically defined as a serum urate concentration exceeding 380 μmol/L in women and 480 μmol/L in men, have been recognized as epidemic metabolic disorder. Beyond its association with gout, hyperuricemia is implicated in various complications such as hypertension, cardiovascular disease, and diabetes, imposing substantial burdens on patient well-being and healthcare costs. While the prevailing interventions predominantly center on renal mechanisms, it is noteworthy that more than one-third of uric acid is eliminated through the gastrointestinal tract (Sorensen, 1965). Presently, emerging insights underscore the gut milieu, particularly the gut microbiota, as an innovative therapeutic avenue, which has captivated researchers exploring new territories in addressing hyperuricemia and its concomitant complications.

Microbiota are increasingly acknowledged as an important part in influencing host development, metabolism, homeostasis, and immunity (Schluter et al., 2020; Cai et al., 2022; Spindler et al., 2022). Exhibiting a myriad of beneficial impacts on the host, microbiota have got substantial attention. Advances in high-throughput sequencing have facilitated a comprehensive profiling of gut microbial compositions and their functional capacities in metabolic disease, including obesity, insulin resistance, and type 2 diabetes (Heianza et al., 2019; Wu H. et al., 2020; Choi and Yang-Jensen, 2023). Their intricate symbiotic association with the host and their potential as therapeutic targets have unveiled their roles in a spectrum of disease. Ongoing research highlights the pivotal involvement of gut microbiota in the etiology of hyperuricemia. Converging evidence suggests distinctive gut microbiota signatures both in gout patients and hyperuricemic rodent models (Lin et al., 2021; Xu et al., 2021). Our prior investigations have further demonstrated that transplanting hyperuricemic fecal microbiota into normal rats elevates serum uric acid levels, evidencing the influential role of microbiota in hyperuricemia development (Liu et al., 2020). Microbiota actively partake in purine metabolism and intricately engage in host-microbe crosstalk. Except for synthesizing purines, gut microbiota can break down dietary purines, converting them into intermediate metabolites, like xanthine and hypoxanthine. Some microbiota also can interconvert purine derivatives. For example, they can convert xanthine to uric acid (Crane, 2013; Wu J. et al., 2020; Grelska et al., 2023). Thus, the intricate interplay between gut microbiota and hyperuricemia remains a subject of growing interest. Despite diligent efforts to ascertain pivotal microbiomes pertinent to hyperuricemia, extant research primarily provides momentary snapshots of the microbiome landscape at single time points. Given the intricate and multifaceted processes underpinning hyperuricemia progression, concurrent and oscillatory microbiota alterations, elusive critical microbiota identification persists. However, knowledge about the dynamic temporal shifts within the gut microbiota during hyperuricemia progress is absent.

Notably, the mechanisms through which gut microbiota exert their influence on disease pathogenesis primarily center on the disruption of intestinal barrier integrity. Abnornal gut microbiota can activated toll-like receptors (TLRs) and nucleotide-binding oligomerization domain-containing proteins (NODs) by increasing detrimental metabolites and reducing beneficinal metabolites such as short-chain fatty acid, and then overproducing proinflammatory cytokines tumor necrosis factor a (TNF-α), Interleukin-1b (IL-1β), and interferon (IFN)-γ, which diminish epithelial barrier function (Capaldo et al., 2012; Wang and Yang, 2022). Impaired intestinal barrier function constitutes a pivotal link between gut microbiota and the host, precipitating pathophysiological cascades across various gastrointestinal and extraintestinal maladies including celiac disease, diabetes, non-alcoholic fatty liver disease (NAFLD), and insulin resistance (Gueddouri et al., 2022). Substantively, over one-third of uric acid excrete through the intestinal tract, and intestinal dysfunction significantly contributes to hyperuricemia (Sorensen, 1965; Hosomi et al., 2012; Pan et al., 2020). Our previous research unveiled hyperuricemia was characteristic by a defective intestinal barrier. Importantly, we found serum uric acid levels exhibited a positive correlation with intestinal permeability, which spotlighted the indispensable role of the intestinal barrier in hyperuricemia development (Lv et al., 2020). Nonetheless, the temporal dynamics of gut barrier alterations during hyperuricemia progression and the intricate interplay between the gut barrier and microbiota remain underexplored. Consequently, finding the pivotal microbiota that underlie compromised intestinal barrier function assumes paramount significance for effective hyperuricemia management.

To furnish a more comprehensive comprehension of the protracted dynamics characterizing gut microbiota and the intestinal barrier throughout the hyperuricemia progress, we conducted a meticulous longitudinal analysis from pre-hyperuricemia to established hyperuricemia in murine models. Employing a comprehensive profiling approach, we investigated the trajectory of gut microbiota, the status of the intestinal barrier, as well as pertinent biochemical indicators during healthy and hyperuricemic states. Additionally, we identified specific microbiota associations with uric acid levels and intestinal barrier. Our study endeavors to extract nuanced insights through intricate data analzes, advancing the identification of innovative therapeutic targets for the prevention and management of hyperuricemia.

## 2 Materials and method

### 2.1 Mouse models

All experiments involving mice were conducted with the approval of the Animal Research Ethics Committee of the Affiliated Hospital of Qingdao University. Male C57BL/6 J mice, 7 weeks old (provided by Beijing Vital River Laboratory Animal Technology Co., Ltd., Beijing, China) acclimated for 1 week prior to experimentation. Mice were randomly divided into two groups: the hyperuricemia group and the control group. The mice were kept under a 12-h light/dark cycle and provided with a standard diet and water *ad libitum*. Each group consisted of five mice. To induce hyperuricemia, the hyperuricemia group (HY) was subjected to intragastric administration of adenine (50 mg/kg) and potassium oxalate (125 mg/kg), diluted in 0.5% carboxymethyl cellulose sodium, on a daily basis for a continuous period of 21 days. The control group received intragastric administration of 0.5% carboxymethyl cellulose sodium alone (NC). Fecal and blood samples were collected at 3, 7, 14, and 21 days after the start of the modeling process. These samples were immediately frozen in liquid nitrogen for subsequent processing.

### 2.2 Biochemical analysis

Serum levels of UA, BUN, CRE, TG, TC, TBA, HDL, and LDL were measured using a 7,020 automatic biochemistry analyzer (Hitachi Ltd., Tokyo, Japan). The levels of serum LPS, TNF-α, and IL-1β were determined using commercial enzyme-linked immunosorbent assay kits from Cusabio Bio-tech Co. Ltd. (Wuhan, Hubei, China), following the manufacturer’s protocol.

### 2.3 Intestinal permeability measurement

The 4-kDa fluorescein isothiocyanate (FITC)-labeled dextran (FD4; Sigma–Aldrich) was used to assess *in vivo* intestinal permeability. After a 4-h water deprivation period, mice were orally administered FD4 (40 mg/100 g body weight) and provided with normal drinking water. After 4 h, serum was collected, and fluorescence intensity was measured with an excitation wavelength of 485 nm and an emission wavelength of 528 nm. FD4 concentration is calculated according to the standard curve. The concentration of FD4 represents intestinal permeability.

### 2.4 Gut microbiome analysis

#### 2.4.1 16S rRNA gene amplification and sequencing

DNA extraction from fecal samples was conducted using the QIAamp Fast DNA Stool Mini Kit. The V3–V4 regions of the 16S rRNA were amplified from metagenomic DNA using specific primers (319f: 5′-AC TCCTACGGGAGGCAGCAG-3′ and 806r: 5′-GGACTACHVGGGTWTCTAAT-3′) to construct an amplicon sequencing library. PCR amplification was performed followed the instructions. The resulting amplicons were purified using the AxyPrep DNA GelExtraction Kit (Axygen Biosciences, Union City, CA, United States), quantified using QuantiFluor-ST (Promega, Madison, WI, United States), and pooled during the cleaning process using magnetic beads from Beckman Coulter. Qualified amplicons underwent paired-end sequencing on an Illumina Novaseq 6,000. The sequencing procedure was carried out by Beijing Biomarker Technology. Raw sequencing data are available in the Sequence Read Archive database under accession number: PRJNA982419.

#### 2.4.2 Amplicon sequence analysis

The raw sequencing data were subjected to filtration using Trimmomatic v0.33. Clean Reads were extracted from the reads by eliminating barcodes and primers with Cutadapt 1.9.1. Reads with low quality (average qual <35 bp) and ambiguous bases were excluded. De-multiplexed sample reads were merged based on overlapping sequences using Usearch v10, discarding reads that could not be merged. Chimeric amplicons were subsequently eliminated using UCHIME v4.2.

Effective reads were further clustered into operational taxonomic units (OTUs) of the 16S rRNA at a 97% similarity threshold using Usearch v10. Taxonomic annotation utilized Ribosomal Database Project (RDP) Classifier v.2.2, referencing the Silva database version 123. Community classification at phylum, class, order, family, genus, and species levels were subsequently validated through NCBI database blasting, and abundance at different taxonomic levels was generated using QIIME software. OTUs constituting less than 0.005% of the total reads were filtered out. Alpha-diversity and beta-diversity analzes were conducted using the R programming package. Anosim (analysis of similarities) was employed to assess differences in beta diversity among distinct groups. The linear discriminant analysis (LDA) effect size (LEfSe) method was used to identify differentially relevant taxa, setting the logarithmic score threshold for LDA analysis at 3.0. Redundancy analysis (RDA), accomplished using the R programming package, illustrated the relationship between bacterial communities and environmental factors. An inter-omic network, constructed using Spearman correlation, provided insight into species interactions within the same environment.

### 2.5 Statistical analysis

Statistical differences among three or more groups were assessed using one-way analysis of variance (ANOVA), followed by Tukey’s multiple comparison posttest. The SAS statistical package facilitated the calculation of non-parametric correlations, particularly the Spearman’s rho, between specific taxa and uric acid or intestinal permeability. The correlation between variables was analyzed using Spearman’s R coefficient. A significance level of *p* < 0.05 was considered indicative of a significant difference.

## 3 Results

### 3.1 The dynamics of uric acid and lipid metabolic during hyperuricemia progress

**Figure 1:**
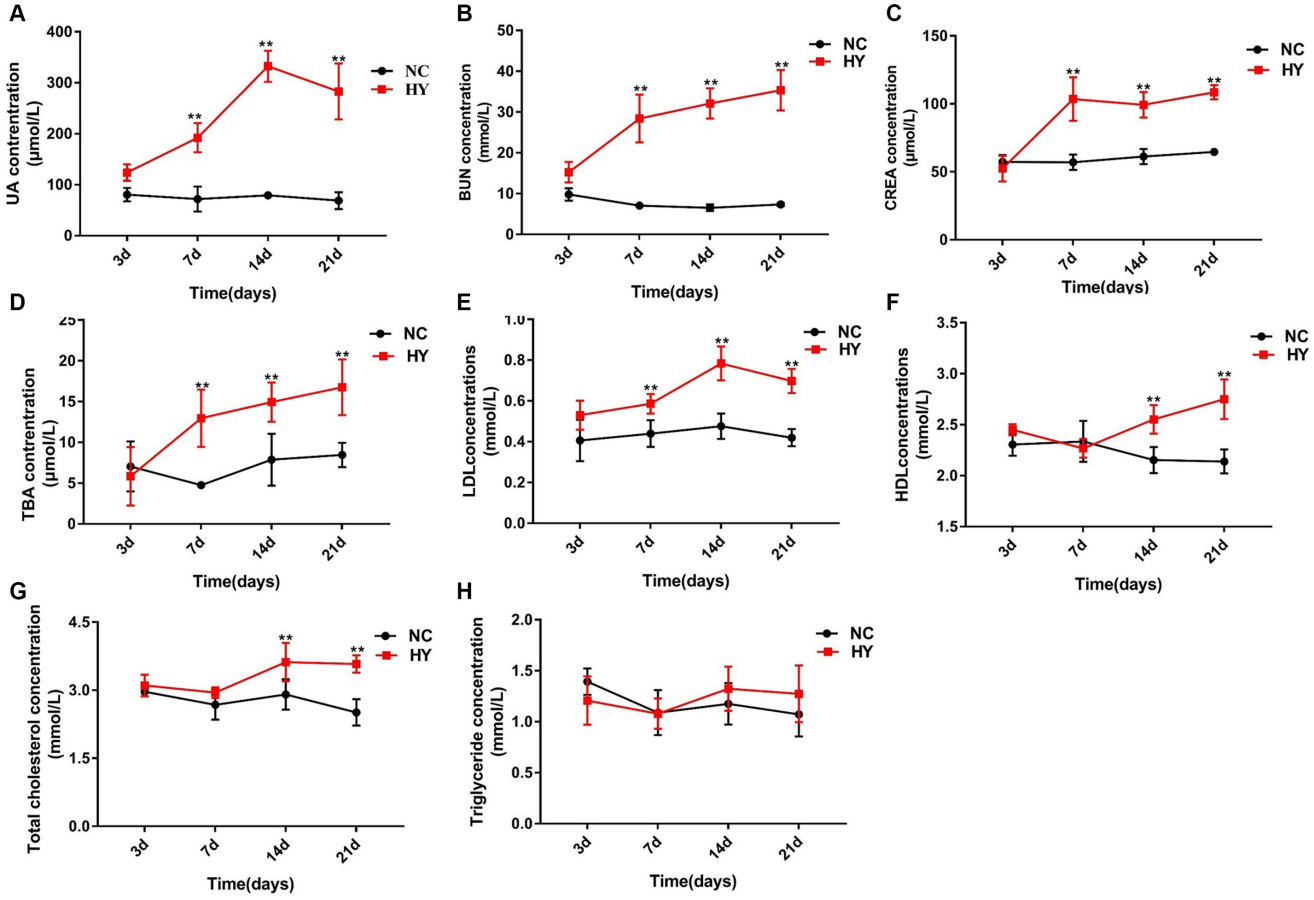
The dynamics of lipid metabolic changes during the progress of hyperuricemic. **(A–F)** The mice was intragastric administration of adenine and potassium oxalate for 21 days (*n* = 5 per group), and blood samples were collected at 3, 7, 14 and 21 days. Serum uric acid **(A)**, BUN **(B)**, CRE **(C)**, TBA **(D)**, LDL **(E)**, HDL **(F)**, TC **(G)** and TG **(H)** levels were detected using an automatic biochemical analyzer. NC, control group; HY, hyperuricemic model mice. Data are presented as the mean ± SEM. Data with different superscript letters are significantly different (*p* < 0.05) using one-way ANOVA followed by Tukey’s multiple comparison post-test (*p < 0.05 and **p < 0.001).

No significant differences in serum UA levels were detected between the NC and HY groups after administering adenine and potassium oxalate for 3 days. However, a dramatic increase in UA levels was observed on the 7th day in the HY group. Subsequently, UA levels exhibited a continuous rise on the 14th day, followed by gradual stabilization on the 21st day (Figure 1A). Correspondingly, levels of BUN and CREA in the HY group significantly increased on the 7th day and remained elevated throughout subsequent stages (Figures 1B,C). These findings indicated the successful establishment of the hyperuricemia model after administering adenine and potassium oxalate for 7 days, sustaining until the 21st day.

We further examined the time-series data of indicators related to lipid metabolism in response to uric acid changes. As depicted in Figures 1D,E, the hyperuricemia group exhibited significantly higher levels of TBA and LDL on the 7th day. Along with the elevation of uric acid, levels of TBA and LDL gradually increased over time. TC levels also experienced elevation on the 14th day and maintained heightened levels on the 21st day (Figure 1G). The aberrant levels of TBA, LDL, and TC signified the presence of dyslipidemia in hyperuricemia mice. TG levels, however, showed no significant changes across time points (Figure 1H). Remarkably, HDL levels in the HY group were significantly higher than those in the NC group on both the 14th and 21st days (Figure 1H).

### 3.2 Dynamics of intestinal permeability and proinflammatory cytokines during hyperuricemia progress

**Figure 2:**
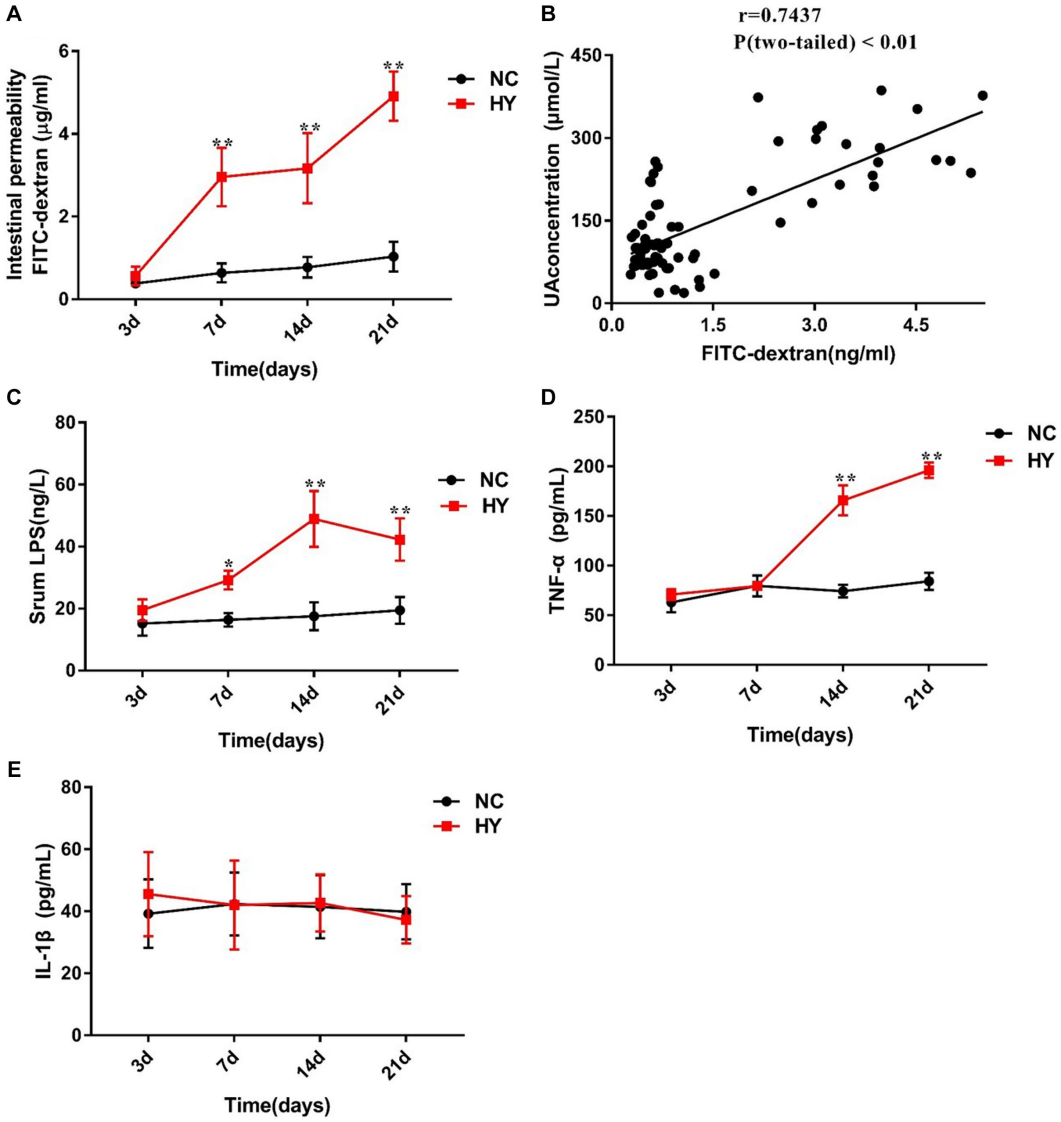
The dynamics of intestinal permeability and proinflammatory cytokines. **(A)** Intestinal permeability was detected in NC and HY mice at 3, 7, 14 and 21 days by administration of FD4, and levels of serum FITC-dextran present intestinal permeability. **(B)** Correlation of intestinal permeability and the uric acid was analyzed using Pearson correlation coefficients. **(C–E)** Serum LPS **(C)**, TNF-α **(D)** and IL-1β **(E)** levels were detected at 3, 7, 14 and 21 days using ELISA. *n* = 5 per group. Data are presented as the mean ± SEM. Data with different superscript letters are significantly different (*p* < 0.05) using one-way ANOVA followed by Tukey’s multiple comparison post-test; *p < 0.05 and **p < 0.001 versus NC.

We proceeded to investigate the dynamics of intestinal permeability by measuring serum FD4 concentration on 3rd, 7th, 14th, and 21st days. In tandem with the increase in UA, intestinal permeability increased significantly in the HY group on the 7th day, which continued to rise on the 14th and 21st days and was higher than NC group. In contrast, the NC group maintained consistently lower intestinal permeability across all time points (Figure 2A). Moreover, we assessed the relationship between UA and intestinal permeability using Pearson correlation analysis. As illustrated in Figure 2B, UA levels demonstrated a strong positive correlation with intestinal permeability (*r* = 0.7437, *p* < 0.01). These findings underscored the fluctuation of intestinal permeability in response to varying UA levels.

Considering that compromised gut barrier integrity leads to the entry of microbial products into the systemic circulation, we further examined the serum levels of LPS, IL-1β, and TNF-α. As anticipated, circulating levels of LPS in HY mice were significantly higher than those in NC mice on the 7th, 14th, and 21st days. A general trend of increased LPS levels over time was observed in the HY mice (Figure 2C), suggesting a potential synchronization between changes in LPS and variations in UA and intestinal permeability. Notably, compared with NC group, serum TNF-α levels in the HY group exhibited a relative increase on the 14th and 21st days, while IL-1β levels showed no significant changes (Figures 2D,E). Importantly, no significant alterations in serum LPS, IL-1β, TNF-α, or intestinal permeability were observed before the onset of hyperuricemia (on the 3rd), confirming the association between hyperuricemia and systemic inflammation.

### 3.3 Dynamics of gut microbiome diversity during hyperuricemia progress

**Figure 3:**
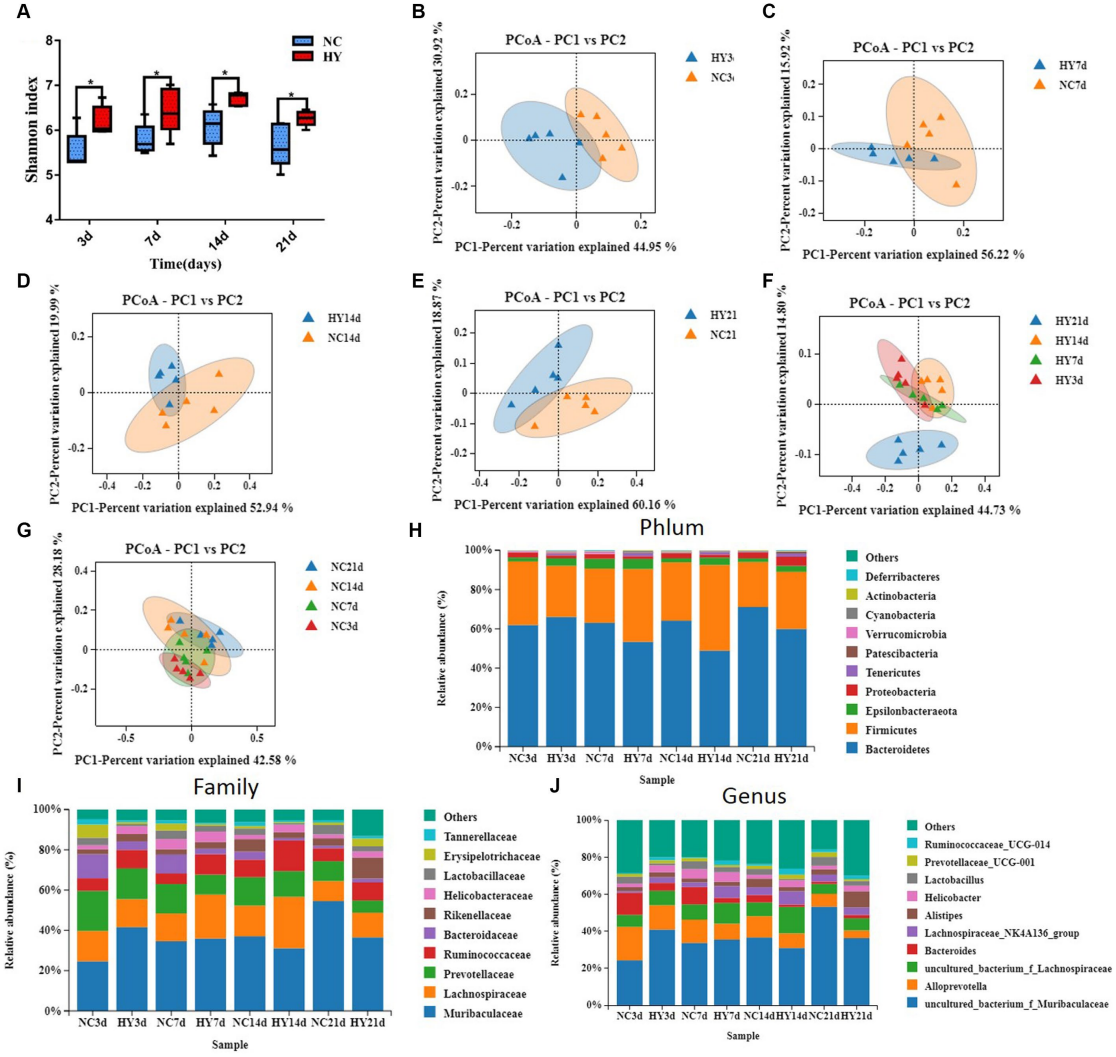
Microbial community structure fluctuated violently in the progress of hyperuricemia. **(A)** Longitudinal tracking of changes in α diversity at 3, 7, 14 and 21 days. **(B–E)** Principal coordinates analysis (PCoA) of gut microbiota composition based on weighted_unifrac for mice before hypeuricemia **(B)** and during the progress of hypeuricemia **(C–E)** compared with NC mice. PCoA1 and PCoA2 represent the top two principal coordinates that captured most of the diversity. The fraction of diversity captured by the coordinate is given as a percentage. **(F,G)** Longitudinal tracking of changes in PcoA of HY **(F)** and NC **(G)** mice at 3, 7, 14 and 21 days. **(H,I)** The relative abundance of top 10 phyla **(H)**, Family **(I)** and genera **(J)** in NC and HY group at 3, 7, 14 and 21 days. Each color blocks represents the relative abundance of a bacterial taxa; *n* = 5 per group.

To comprehend the dynamic changes in microbial diversity, we analyzed the alpha diversity (Shannon index) and beta diversity of the gut microbiome. In comparison to the NC mice, the HY group consistently displayed significantly increased Shannon index values at all time points, even during the pre-hyperuricemia stage. However, within the HY group, no significant changes in alpha diversity were observed over time, starting from the pre-hyperuricemia period (Figure 3A). Principal coordinate analysis (PCoA) plots based on weighted_unifrac distance demonstrated distinct clustering of NC and HY groups on 3rd, 7th, 14th, and 21st days, illustrating marked differences in microbiota community membership and structure (Figures 3B–E). Intriguingly, hyperuricemia elicited a notable alteration in gut microbial composition within the HY group over time. Particularly, on 21st day, the microbial community structure markedly diverged from other time points, while microbiomes in the NC group maintained similar trajectories at various time points (Figures 3F,G). Moreover, analysis of the top 10 most prevalent phyla, families, and genera through taxonomy profiling revealed dysbiosis and heterogeneity in the gut microbiota composition of the HY group at different time points compared to the NC group (Figures 3H–J).

### 3.4 Dynamics of distinct gut microbiome during hyperuricemia progress

**Figure 4:**
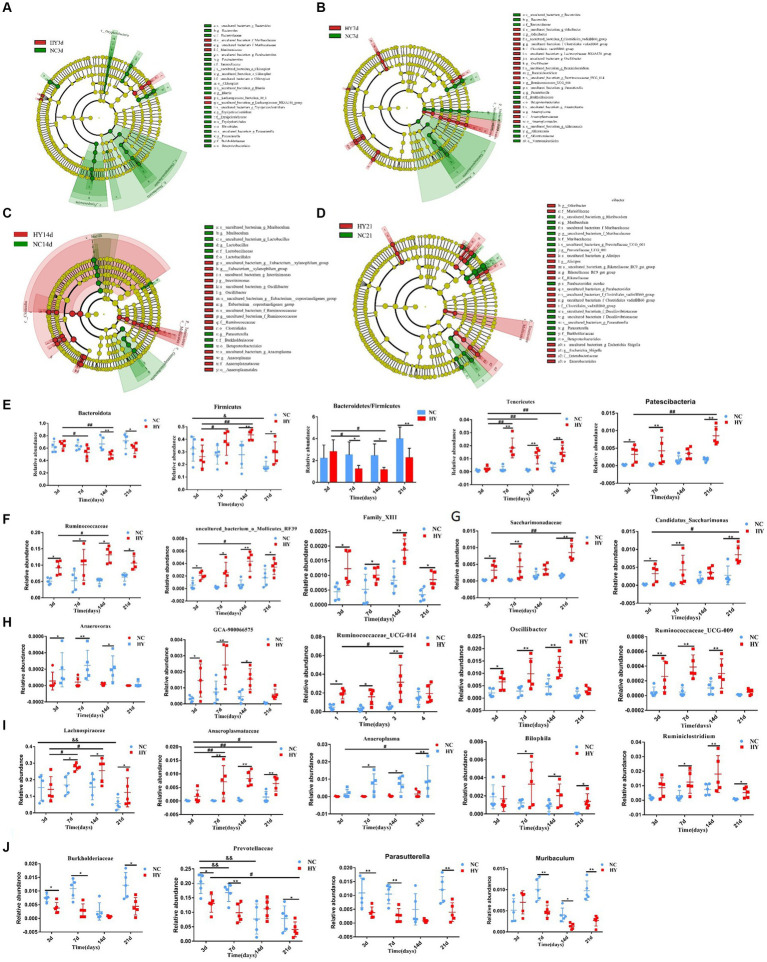
The dynamics of distinct gut microbiome. **(A–D)** The cladogram was analyzed using A linear discriminant analysis effect size (LEfSe) to represent diferentially abundant taxa from NC (blue) and HY (red) at 3 **(A)**, 7 **(B)**,14 **(C)**, and 21 days **(D)** Only taxa with a statistically significant LDA score > 3.5 was showed. Each node denotes a taxonomic unit within the bacterial hierarchy and nodes of different colors represent the crucial microbiome. Diameter of the small circle is proportional to the relative abundance. **(E)** The persist increased or decreased taxa at phylum in the progress of hyperuricemia. **(F)** The family and genera that persist increased from 3 days to 21 days. **(G)** The family and genera that increased at 3, 7 and 21 days. **(H)** The family and genera that increased at 3, 7 and 14 days. **(I)** The family and genera that increased at 7, 14 and 21 days. **(J)** The family and genera that decreased at 3, 7and 21 days; *n* = 5 per group. Data are presented as the mean ± SEM. Data with different superscript letters are significantly different (*p* < 0.05) using one-way ANOVA followed by Tukey’s multiple comparison post-test; *p < 0.05 and **p < 0.001 versus NC; ^#^ *p* < 0.05 and ^# #^ *p* < 0.001 versus 3d.

To identify specific microbiota contributing to hyperuricemia, we performed linear discriminant analysis effect size (LEfSe) with LDA scores >3.5. We found hyperuricemia induced diverse changes in gut microbial composition across different stages. During the pre-hyperuricemia phase, 26 differentially abundant bacteria were observed between the NC and HY groups. Compared to the NC group, the HY group exhibited elevated levels of 5 bacteria, including the *Muribaculaceae* family, *uncultured_bacterium _f_Muribaculaceae* genus, and *lachnospiraceae_bacterium_10_1* species. In contrast, 21 bacteria, including *Bacteroides*, *Parabacteroides*, *Blautia*, *Erysipelatoclostridium*, *Parasutterella*, *Bacteroidaceae*, *Tannerellaceae*, *Erysipelotrichaceae*, *Burkholderiaceae*, *Chloroplast*, Erysipelotrichales, Rhizobiales, and Betaproteobacteriales, were reduced (Figure 4A). Upon an increase in uric acid levels on 7th day, 27 differentially abundant bacteria were identified, with 16 being elevated and 11 reduced. Notably, pathogenic bacteria associated with infection and inflammation, such as *Odoribacter*, *Oscillibacter*, and *Anaeroplasmatales*, were significantly increased in the HY group, while beneficial bacteria, including *Bacteroides*, Akkermansia, Bacteroidaceae, and Akkermansiaceae, were significantly diminished (Figure 4B). Elevated uric acid levels sustained on 14th day, and corresponding alterations persisted. Compared to the NC group, increased abundance was observed in *Eubacterium_xylanophilum_group*, *Intestinimonas*, *Oscillibacter*, *Eubacterium_coprostanoligenes_group*, *Anaeroplasma*, *Ruminococcaceae*, *Anaeroplasmataceae*, *Clostridiales*, and *Anaeroplasmatales*, while *Muribaculum*, *Lactobacilus*, *Parasutterella*, *Lactobacillaceae*, *Burkholderiaceae*, *Lactobacillales*, and *Betaproteobacteriales* were reduced (Figure 4C). More pronounced changes were evident on 21st day. Abundance of *Odoribacter*, *Alistipes*, *Rikenellaceae_RC9_gut_group*, *Escherichina_Shigella*, *Marinifilaceae*, *Rikenellaceae*, *Clostridiales_vadinBB60_group*, *Enterobacteriaceae*, and *Enterobactericles* was higher, while *Muribaculum*, *Prevotellaceae_UGG_001*, *Parasutterella*, *Parabacteroides_merdae*, *Muribaculaceae*, *Burkholderiaceae*, and *Betaproteobacteriales* were reduced in the HY group (Figure 4D). Most of the significant changes in microbiota composition within specific time intervals were transient, except for the genera *Parasutterella*, family *Burkholderiaceae*, and Order *Betaproteobacteriales*, which persisted on 21st day.

We also evaluated specific bacteria that exhibited substantial and lasting changes during the progression of hyperuricemia. ANOVA was employed for this analysis, focusing on markers with monotonically increasing or decreasing trends over time. At the phylum level, *Bacteroidota* and *Firmicutes*, the predominant microbiota, demonstrated significant changes. In comparison to the NC group, *Bacteroidota* levels were reduced, while *Firmicutes* levels were elevated in the HY group on both 14th and 21st days. However, the abundances of Bacteroidota and Firmicutes were comparable between the NC and HY groups on 3rd and 7th days. Within the HY group, Bacteroidota decreased, and Firmicutes increased on 7th and 14th days when compared to the pre-hyperuricemia stage (3rd day). Notably, the Bacteroidota/Firmicutes ratio was significantly lower on 7th, 14th, and 21st days following the rise in uric acid levels. This ratio also demonstrated a declining trend within the HY group. No changes were observed within the NC group. In contrast, *Tenericutes* and *Patescibacteria* increased consistently within the HY group (except on 3rd day for Tenericutes and 14th day for Patescibacteria) (Figure 4E). At the family and genus levels, increased abundance was noted for family Ruminococcaceae, uncultured_bacterium_o_Mollicutes_RF39, and Family_XIII from 3rd to 21st days in the HY group (Figure 4F). Family Saccharimonadaceae and genus Candidatus_Saccharimonas also exhibited increased abundance at all stages except on 14th days (Figure 4G), whereas genus Anaerovorax, GCA-900066575, Ruminococcaceae_UCG-014, Oscillibacter, and Ruminococcaceae_UCG-009 displayed increased abundance on 3rd, 7th, and 14th days but not on 21st day (Figure 4H). In contrast, family *Lachnospiraceae*, *Anaeroplasmataceae*, and genera *Anaeroplasma*, *Bilophila*, and *Ruminiclostridium* increased concomitantly with uric acid levels (Figure 4I). Conversely, family *Burkholderiaceae*, *Prevotellaceae*, and genera *Muribaculum* and *Parasutterella* were significantly reduced (Figure 4J). These findings indicated that certain bacteria initiated and exerted persistent effects during hyperuricemia progression.

### 3.5 Correlation analysis of gut microbiota with uric acid and intestinal permeability

**Figure 5:**
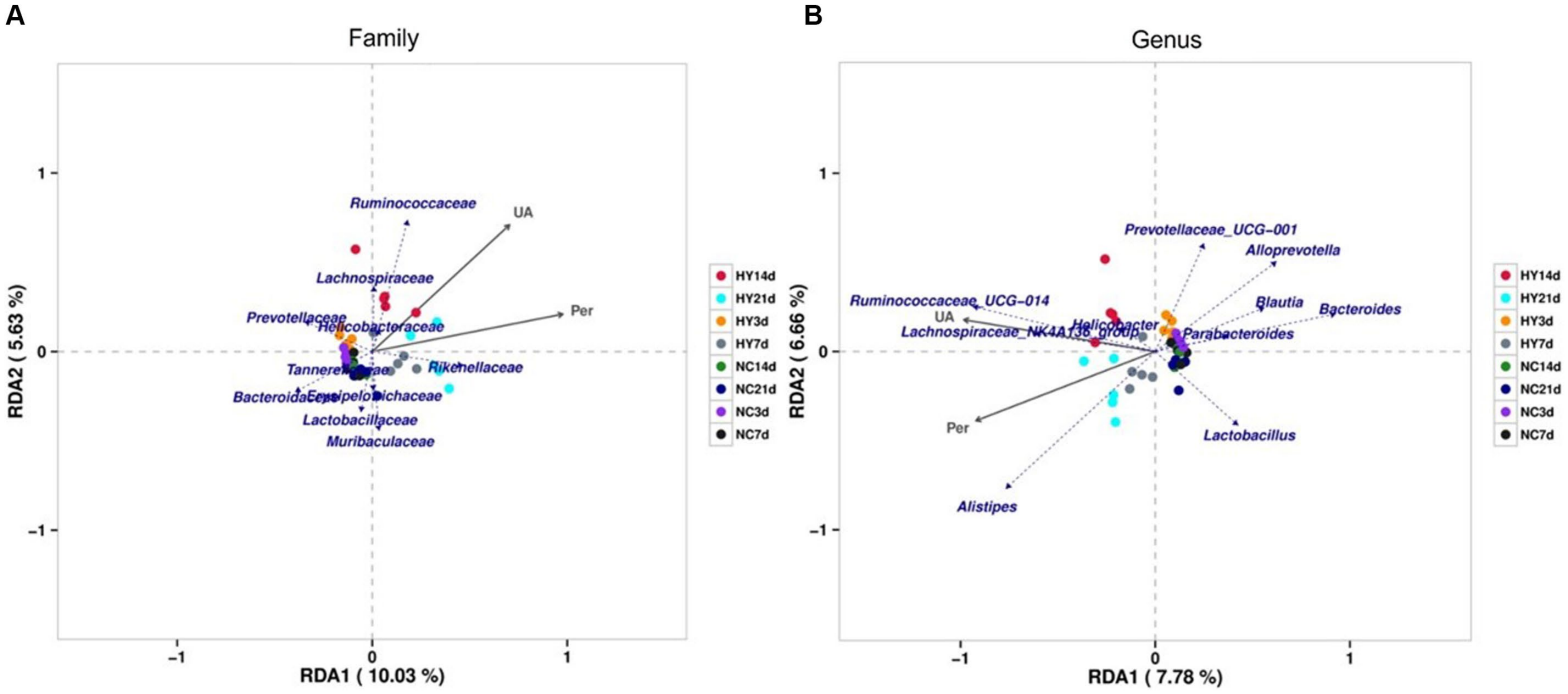
RDA analysis of correlation between gut microbiome, uric acid and intestinal permeability. **(A,B)** The relationship between gut microbiome, uric acid and intestinal permeability at family **(A)** and genus **(B)** using Redundancy analysis (RDA). The top 10 most abundant microbiota was analyzed. The scale on the horizontal and vertical coordinates is the regression analysis calculation with environmental factors. Obtuse angles represent negative correlations and acute angles represent positive correlations; UA, uric acid; Per, intestinal permeability.

We proceeded to identify specific gut microbiota associated with uric acid levels and intestinal permeability through redundancy analysis (RDA). As demonstrated in Figures 5A,B, among the top 10 most abundant microbiota, families *Ruminococcaceae*, *Lachnospiraceae*, *Rikenellaceae*, and *Helicobacteraceae* were positively correlated with both uric acid levels and intestinal permeability. In contrast, families *Tannerellaceae*, *Bacteroidaceae*, *Erysipelotrichaceae*, *Lactobacillaceae*, and *Muribaculaceae* were negatively correlated. Notably, genera *Prevotellaceae_UCG_001*, *Alloprevotella*, *Blautia*, *Bacteroides*, *Parabacteroides*, and *Lactobacillus* exhibited negative correlations, while genera *Ruminococcaceae_UCG_014*, *Lachnospiraceae_NK4A136_group*, *Helicobacter*, and *Alistipes* were positively correlated with uric acid levels and intestinal permeability. Significantly, family *Ruminococcaceae*, *Lachnospiraceae* and genera *Ruminococcaceae_UCG_014* that positively related with uric acid and intestinal permeability were also significantly increased in the progress of hyperuricemia, indicating that the microbiota may dominate hyperuricemia progress and involved in intestinal barrier dysfunction. Also note that, bacteria that correlate with uric acid levels were also association with intestinal permeability, which was in accordance with the results that there was a strong correlation between uric acid and intestinal permeability as already described.

Pearson’s correlation analysis was further employed to assess the significant correlations between microbial communities, uric acid levels, and intestinal permeability. Only abundances above 0.1% were analyzed. A total of 11 families and 17 genera exhibited significant correlations with uric acid levels, while 13 families and 21 genera correlated with intestinal permeability (Tables 1 2). Notably, family *Burkholderiaceae* and genus *Parasutterella* were strongly inversely correlated with UA (R < −0.5, *p* < 0.01), while family *Anaeroplasmataceae*, *uncultured_bacterium_o_Mollicutes_RF39*, *Clostridiales_vadinBB60_group*, *Saccharimonadaceae*, *Ruminococcaceae*, *Family_XIII*, and genera *Anaeroplasma*, *Candidatus_Saccharimonas*, and *Ruminococcaceae_UCG-014* were significantly positively correlated with UA (R > 0.5, *p* < 0.01). Significant negative correlations existed between intestinal barrier and protective bacteria including Family *Akkermansiaceae*, genus *Akkermansia* and SCFA produced bacteria including family *Bacteroidaceae*, genus *Bacteroides* (R < -0.5, p < 0.01), while family *Anaeroplasmataceae*, *uncultured_bacterium_o_Mollicutes_RF39*, *Clostridiales_vadinBB60_group*, *Saccharimonadaceae*, *Eggerthellaceae* and genus *Anaeroplasma*, *Candidatus_Saccharimonas*, *Ruminocooccaceae_UCG-014*, *Desulfovibrio*, *Enterorhabdus* positively correlated with intestinal barrier (R > 0.5, *p* < 0.01). Moreover, several families and genera exhibited strong correlations with intestinal permeability and uric acid levels, reflecting the intricate interplay between gut microbiota, uric acid levels, and intestinal barrier function.

**Table 1:** Correlation of gut microbiome between uric acid and intestinal permeability at genus levels.

Gut bacteria	Uric acid		Intestinal Permeability	
	R value	*p* value	R value	p value
Parasutterella	−0.695	0.000	−0.429	0.006
Akkermansia	−0.481	0.002	−0.680	0.000
Muribaculum	−0.492	0.001	−0.342	0.031
Parabacteroides	−0.389	0.013	−0.432	0.005
Bacteroides	−0.327	0.039	−0.571	0.000
Coprobacillus	−0.310	0.052	−0.511	0.001
Lachnoclostridium	−0.247	0.125	−0.572	0.000
Alloprevotella	−0.233	0.148	−0.420	0.007
Uncultured_bacterium_o_Mollicutes_RF39	0.680	0.000	0.674	0.000
Anaeroplasma	0.630	0.000	0.684	0.000
Candidatus_Saccharimonas	0.565	0.000	0.638	0.000
Uncultured_bacterium_f_Clostridiales_vadinBB60_group	0.565	0.000	0.679	0.000
Uncultured_bacterium_f_Ruminococcaceae	0.503	0.001	0.098	0.547
Ruminococcaceae_UCG-014	0.522	0.001	0.532	0.000
Ruminococcaceae_UCG-005	0.471	0.002	0.421	0.007
Oscillibacter	0.468	0.002	0.101	0.535
[Eubacterium]_xylanophilum_group	0.465	0.003	0.392	0.012
Ruminiclostridium	0.451	0.003	0.275	0.086
Desulfovibrio	0.437	0.005	0.577	0.000
Rikenellaceae_RC9_gut_group	0.401	0.010	0.363	0.021
Odoribacter	0.297	0.063	0.472	0.002
Uncultured_bacterium_f_Erysipelotrichaceae	0.242	0.133	0.550	0.000
Roseburia	0.213	0.187	0.405	0.009
Enterorhabdus	0.216	0.182	0.652	0.000

Correlation between variables was analyzed by Spearman’s R coefficient. R represents a linear correlation between the two variables. The closer the absolute value of. r is to 1, the stronger the linear correlation between the two variables. R > 0 is positive correlation, R < 0 is negative correlation. *p* < 0.05 was considered a significant difference.

**Table 2:** Correlation of gut microbiome between uric acid and intestinal permeability at family levels.

Gut bacteria	Uric acid		Intestinal Permeability	
	R value	*p* value	R value	*p* value
Burkholderiaceae	−0.684	0.000	−0.414	0.008
Akkermansiaceae	−0.481	0.002	−0.680	0.000
Tannerellaceae	−0.389	0.013	−0.432	0.005
Bacteroidaceae	−0.327	0.039	−0.571	0.000
Deferribacteraceae	−0.174	0.283	−0.370	0.019
Prevotellaceae	−0.159	0.328	−0.393	0.012
Anaeroplasmataceae	0.630	0.000	0.684	0.000
Uncultured_bacterium_o_Mollicutes_RF39	0.680	0.000	0.674	0.000
Clostridiales_vadinBB60_group	0.565	0.000	0.679	0.000
Saccharimonadaceae	0.565	0.000	0.638	0.000
Ruminococcaceae	0.534	0.000	0.321	0.043
Family_XIII	0.523	0.001	0.263	0.102
Rikenellaceae	0.329	0.038	0.350	0.027
Eggerthellaceae	0.216	0.182	0.652	0.000

Correlation between variables was analyzed by Spearman’s R coefficient. R represents a linear correlation between the two variables. The closer the absolute value of. r is to 1, the stronger the linear correlation between the two variables. R > 0 is positive correlation, R < 0 is negative correlation. *p* < 0.05 was considered a significant difference.

### 3.6 Altered bacterial correlation network in hyperuricemia mice

**Figure 6:**
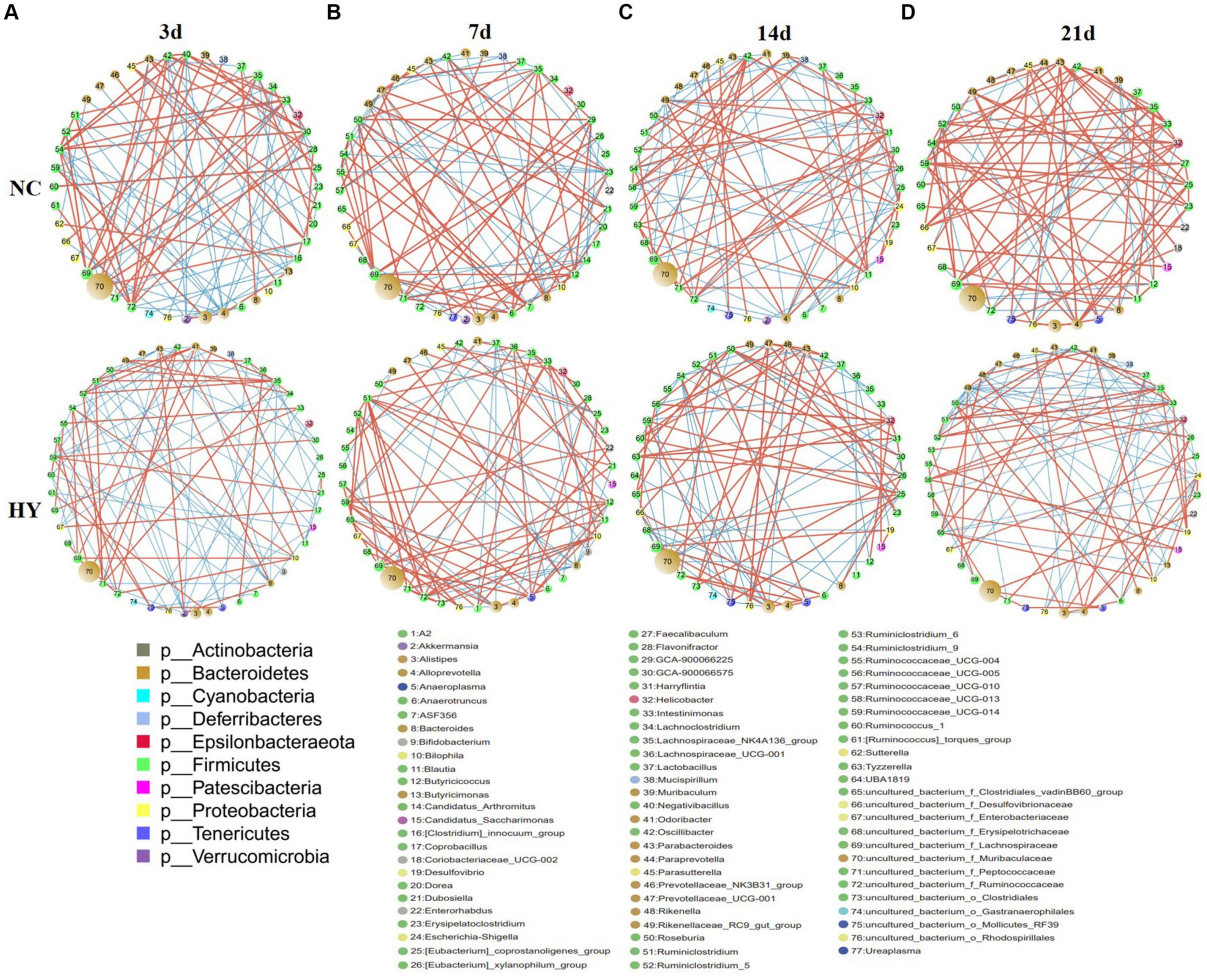
Disrupt bacterial network in the progress of hyperuricemia. **(A–D)** Networks in NC and HY at 3 **(A)**, 7 **(B)**, 14 **(C)** and 21 days **(D)** were performed using Cytoscape. Each circles represents a microbial genus. The size of the circle represents the abundance of the moacobiota and the color representing genera refers to phylum as indicated. The lines represent the correlation between the two genera and the thickness is proportional to correlation coefficients. Red and Blue indicates positive and negative correlations, respectively.

To delve into the microbial ecosystem structure, we constructed a correlation network at the genus level using the SparCC algorithm. As depicted in Figures 6A–D, the network in the NC group exhibited a stable and dense structure with positive and negative correlations distributed across nodes, displaying moderate fluctuations over time. In contrast, the networks in the HY group differed significantly, characterized by a loosely connected structure, notable node attractions, and cumulative strong correlations among specific bacteria. Hubs, defined as bacteria connected to at least eight genera, exhibited no significant difference in numbers between the NC and HY groups on 3rd and 7th days. However, with increasing uric acid levels, the number of hubs in the HY group dramatically decreased on 14th and 21st days (10 and 9 hubs in NC, and 6 and 5 hubs in HY group on 14th and 21st days, respectively), indicating a fragile microbial community structure in the HY group. Notably, most of the hubs in the NC group belonged to the Firmicutes phylum, whereas those in the HY group mainly belonged to the *Bacteroidetes* and *Firmicutes* phyla. Interestingly, *uncultured_bacterium_f_Lachnospiraceae* served as a hub at various stages in the NC group, while it was absent in the HY group.

We further identified positive and negative correlation hubs (bacteria positively or negatively connected to at least seven genera). On 3rd day, the NC group harbored four positive correlation hubs, including *Ruminiclostridium_9*, *Intestinimonas*, *uncultured_bacterium_f_Lachnospiraceae*, and *uncultured_bacterium_f_Ruminococcaceae*. No negative hubs were detected. Conversely, the HY group featured three negative hubs, namely *Parabacteroides*, *Akkermansia*, and *Bacteroides*, with strong positive correlations between these hubs. Notably, all of the negative hubs were both negatively correlated with *Ruminiclostridium_5* and *Ruminiclostridium*, the bacteria that increased in HY group from 14th and 7th, respectively (Figure 6A).

There is one positive and one negative hub in NC while two positive and one negative hubs in HY group at 7d. In HY group, the two positive hubs *Ruminiclostridium* and *uncultured_bacterium_f_Ruminococcaceae* were negatively correlated with the negative hub *Bacteroides*, but positively correlated with *Ruminiclostridium_5*, indicating mutually reinforcing relationship with *Ruminiclostridium_5* but inhibition with *Bacteroides* (Figure 6B). On 14th day, a notable divergence was observed between the NC and HY groups. No positive or negative related hubs were found in NC group, while two negative hubs, including *Ruminiclostridium_9* and *uncultured_bacterium_f_Muribaculaceae* were found in HY group. Genera *Helicobacter* and *Ruminococcaceae_UCG-005* that increased in HY exhibited strong negative correlations with the negative hubs *uncultured_bacterium_f_Muribaculaceae* (Figure 6C). On 21st day, the differences between the two groups intensified. In the NC group, nine positive correlation hubs were identified, indicating that the bacteria were mutual promotion to build a stable ecosystem. Conversely, the HY group harbored two negative correlation hubs, including *Rikenellaceae_RC9_gut_group* and *Escherichia-Shigella*. The two negative correlation hubs were negatively related with 20 genera (Figure 6D), indicating the microbiota in HY group were reciprocal inhibition to build a fragile flora ecology.

## 4 Discussion

The study presented in this work addresses a significant gap in our understanding of the role of gut microbiota in hyperuricemia progression. Unlike most previous research that has provided static snapshots of the microbiome at specific time points, our study presents a unique longitudinal dataset that enables a systematic exploration of the dynamic changes in both gut microbiota and intestinal barrier function throughout the course of hyperuricemia. This approach allowed us to identify key microbiota contributing to hyperuricemia and intestinal barrier dysfunction. The insights gained from this study offer valuable information for understanding the contribution of gut microbiota to the development of hyperuricemia and its associated compromised intestinal barrier, thereby paving the way for the development of novel strategies to mitigate the risk of hyperuricemia-related complications.

Microbiota are complex and heterogeneous entities with dynamic structures and functions. Under normal physiological conditions, microbial communities exhibit stability, but they can undergo rapid and substantial changes in response to minor perturbations (Cho et al., 2012; Faith et al., 2013; David et al., 2014). Our study showed that bacterial diversity and structure was different during the progress of hyperuricemia. Notably, Shannon index values in HY group significantly increased at all time points. However, the results was inconsistent with the study of Song et al. (2022). Song et al. (2022) showed Shannon index values in HY group was slight decreased. The difference may be caused by the different methods in constructing hyperuricemia model. Increased Shannon index in HY group indicated higher community diversity as well as disturbance and uncertainty of bacteria, which was consistent with our results that the elevated flora is increased. The transient alterations in microbiota composition at the early stages of disease may underlie long-term health consequences. For instance, a longitudinal study in individuals with prediabetes revealed changes in gut microbiota at the earliest stages of diabetes, with individuals exhibiting an increased abundance of microbiota positively correlated with impaired glucose tolerance being more likely to develop diabetes later on (Zhou et al., 2019). Similarly, early-life exposure to antibiotics can disrupt the microbiota, impair immune development, and lead to long-term adverse health effects (Guittar and Shade, 2019; Depner et al., 2020; Henrick et al., 2021; Thänert and Thänert, 2021). These studies underscore the importance of early-stage compositional and functional changes in the microbiome as critical windows for disease development. Although previous research has highlighted the microbial signatures associated with hyperuricemia (Liu et al., 2020; Lv et al., 2020; Lin et al., 2021; Xu et al., 2021; Ul-Haq et al., 2022), less attention has been paid to the microbiota changes occurring in the early stages, which may provide incomplete or inadequate interpretations of the microbial community’s state and properties. Our study uniquely reveals that microbiota changes occur before the onset of hyperuricemia. Specifically, we observed a reduction in the abundance of families *Bacteroidaceae* and genus *Bacteroides*, which are major constituents of the gut bacteriome responsible for providing nutrients and vitamins to both the host and other intestinal microbial residents. This sustains the thriving of bacterial communities and helps maintain a healthy ecological environment (Bergstrom and Xia, 2013; Zafar and Saier, 2021; Yu et al., 2023). Similarly, *Blautia* is a dominant probiotic genus known for its involvement in biotransformation and interaction with other intestinal microbiota (Laverde Gomez and Mukhopadhya, 2019; Liu and Mao, 2021). Our findings indicate that the reduced levels of *Bacteroides*, *Bacteroidaceae*, and *Blautia* at the earliest stages of hyperuricemia may lead to disrupted microbiota relationships, exacerbating microbial dysbiosis as hyperuricemia progresses. Furthermore, the reduction of *Parasutterella*, which has been implicated in bile acid maintenance and cholesterol metabolism (Ju et al., 2019), at the earliest stage could potentially contribute to the dyslipidemia associated with hyperuricemia. The short chain fatty acids secreting genus *Parabacteroides* was showed reduce neutrophil infiltration and exert anti-proinflammatory signaling (Wu T. et al., 2019; Lei et al., 2021; Cui et al., 2022). Depletion of *Erysipelotrichales* has also been reported previously in inflammatory bowel disease dog (Díaz-Regañón et al., 2023) and crohn disease (Gevers et al., 2014). Notably, the reduction of key bacterial populations at the early stages of hyperuricemia, although not directly linked to abnormal uric acid levels and intestinal barrier function, may initiate a cascade of events that hinder immune development, exacerbate microbiota disruption, and lead to various long-term adverse outcomes.

To better understand the interplay between microbiota and external stimuli, we conducted an interactive analysis of the dynamic changes in bacterial communities and intestinal barrier function during hyperuricemia progression. Notably, we observed a prominent increase in uric acid levels in conjunction with intestinal barrier dysfunction starting from day 7. Coinciding with these changes, the bacterial community also exhibited considerable variability. It is well-established that compromised intestinal barrier function and disrupted microbial communities often occur in tandem, as these factors can mutually influence each other. The gut microbiome plays a pivotal role in the development of intestinal immune responses and the maintenance of intestinal barrier integrity. Depletion of commensal bacteria can create an opportunity for the proliferation of pathogenic bacteria, which can generate harmful metabolites, trigger innate and adaptive immune responses, and ultimately compromise the integrity of the intestinal barrier (Sartor, 2010). In line with this understanding, our study identified reductions in beneficial bacteria such as *Parabacteroides*, *Akkermansia*, and *Bacteroides* on 3rd day, and these reductions were negatively correlated with genera such as *Ruminiclostridium_5*, *Ruminiclostridium*, suggesting a competitive microbial community dynamic being established. Additionally, we noted the persistence of reduced levels of short-chain fatty acid-producing genera such as *Prevotellaceae* and *Muribaculum* throughout hyperuricemia. Conversely, pathogenic bacteria like *Oscillibacter*, which is positively correlated with IL-1β levels and increased in conditions such as ulcerative colitis (Wang et al., 2019; Wu M. et al., 2019), exhibited increased abundance on 3rd day of hyperuricemia. Similarly, the increased presence of the Ruminiclostridium genus, which has been associated with high-fat diets and DSS-induced colitis (Yue et al., 2019; Jo et al., 2021; Qiao et al., 2022), was also observed on 7th, 14th, and 21st days of hyperuricemia. Importantly, our analysis highlighted that Ruminiclostridium, acting as a positive hub, was positively correlated with *Ruminiclostridium_5*, *Intestinimonas*, *uncultured_bacterium_f_Ruminococcaceae*, and *Bilophila*, while being negatively correlated with the presence of *Bacteroides*. *Bilophila* has been shown to induce inflammation and metabolic disorders by restoring and transforming sulfites into hydrogen sulfide (David et al., 2014; Xing et al., 2019; Zhao et al., 2022). *Kivenson V. el al* found *Bilophila* have genomic signatures for genetic code expansion that could enable them to metabolize both trimethylamine (TMA) and its precursors without production of trimethylamine N-oxide (TMAO), which result in bile acid metabolism disorder (Kivenson and Giovannoni, 2020). Bilophila and levels of TBA and LDL increased simultaneously on 7th day and sustained throughout hyperuricemia stages, indicating that *Bilophila* may be involved in bile acid metabolism in hyperuricemia. *Ruminiclostridium_5* and *Intestinimonas* were showed significantly correlated with intestinal permeability and serum LPS (Li Q. et al., 2022; Zhang et al., 2022). Consistently with previous study, combined with the elevated of *Ruminiclostridium_5* and *Intestinimonas*, we found serum LPS and TNF-a was also elevated in hyperuricemia mice. Gut microbiomes have been theorized to be environments with great ecological opportunity, pathogenic organisms can exploit community instability inherent in ecological opportunity to drive pathogen adaptation and intrude (Scanlan, 2019). This intricate network suggests that the influx of *Ruminiclostridium* may create ecological niches favoring the expansion of pathogenic bacteria and suppressing beneficial bacteria, ultimately contributing to increased intestinal barrier dysfunction and uric acid levels. The microbiota correlation network became even more loosely and rickety on 21st day. We found the elevated genera *Rikenellaceae_RC9_gut_group* and *Escherichina_Shigella* were negatively connected with most of genra, indicating gut microbial community shift toward a reciprocal inhibitory state. Expansion of *Escherichia-Shigella* was showed associate with the activation of NLRP3 and high levels of TNF-α and interleukin 6 (Li et al., 2020; Li S. et al., 2022). The increased *Ruminiclostridium_5*, *Intestinimonas*, *Enterobacteriaceae* and *Escherichina_Shigella* may be contributed to the elevated serum LPS and TNF-a. Increased levels of circulating LPS and TNF-a has also been reported in our previous study in UOX−/− mouse (Lv et al., 2020), which likely facilitate illustrate the mechanism that hyperuricemia was associated with metabolic dysfunction such as insulin resistance and atherosis. More intriguingly, except for the transient microbiota changes at different stages, we found a lower persisted ratio of *Bacteroidota/Firmicutes* throughout hyperuricemia. Similarly results was also observed in obese and NAFLD mice with compromised intestinal barrier (Ley et al., 2006; Guo et al., 2023). As a dominant phyla, these taxonomic changes indicating microbiota undergo tremendous community succession and the environment shift toward a proinflammatory state. This ecosystem shift, involving the expansion of opportunistic pathogens like *Ruminiclostridium_5*, *Intestinimonas*, and *Bilophila*, could further facilitate the elevated serum LPS and TNF-α levels observed in hyperuricemia, in turn fueling metabolic inflammation and associated disturbances.

The observed persistent decline in *Burkholderiaceae* and *Parasutterella* was strongly inversely related to uric acid levels, while *Ruminococcaceae* and *Family_XIII* were positively correlated. Remarkably, a similar negative relationship with uric acid was also found in a uricase-knockout hyperuricemia mouse model. *Burkholderiaceae* is known to be involved in the degradation of toluene, and the genus *Burkholderia thailandensis* possesses a major facilitator transport regulator responsible for uric acid degradation (Grove, 2010), hinting at its potential role in urate metabolism. Dysbiosis characterized by an increased proportion of microbiota containing LPS and a decrease in SCFA-producing microbiota can favor the utilization of colonic mucus as a nutrient source, exacerbating pathogen infection and increasing mucus layer permeability. Notably, the relationship between the microbiota and intestinal barrier function has been less explored in the context of hyperuricemia. In our study, we observed that families *Akkermansiaceae* and *Bacteroidaceae* were negatively correlated with intestinal permeability, whereas LPS-containing microbiota such as *Desulfovibrio* and *Enterorhabdus* exhibited positive correlations. This finding aligns with previous studies that have demonstrated decreased levels of *Akkermansiaceae* and *Bacteroidaceae* in obese mice with compromised intestinal barrier function (Clarke et al., 2014; Liu et al., 2021). Intriguingly, several bacteria that persistently increased during hyperuricemia exhibited correlations with both uric acid levels and intestinal permeability, potentially explaining the observed association between uric acid and intestinal permeability. This positive relationship between uric acid and intestinal permeability, previously demonstrated in our study (Lv et al., 2020), was consistently observed throughout hyperuricemia. Intestinal barrier dysfunction is believed to be a significant driver of metabolic syndrome, as pathogenic microbes and their products can access the systemic circulation through a compromised barrier, migrate to inflamed sites, and directly contribute to inflammation in key metabolic tissues, ultimately promoting metabolic inflammation and exacerbating metabolic disturbances (Fizanne et al., 2023; Violi et al., 2023). Given these findings, targeting the intestinal barrier presents an attractive approach for mitigating the metabolic dysfunctions associated with hyperuricemia.

## 5 Conclusion

Our study provided a dynamic landscape of intestinal bacteria throughout hyperuricemia progression. We demonstrated that changes in microbial community structure occur prior to the onset of hyperuricemia and follow distinct developmental phases during the disease course, characterized by the expansion of pathogenic bacteria, reduction of beneficial bacteria, and fragility of the ecological network. Meanwhile, compromised intestinal barrier occurred with elevated uric acid and intestinal permeability progressively increases as the disease progresses. Our study suggested that Ruminiclostridium, Bacteroides, Akkermansiaceae, Bilophila, Burkholderiaceae and Parasutterella were the key bacteria that play vital rols in the progress of hyperuricemia and compromised intestinal barrier. However, in this study, we did not know whether the compromised intestinal barrier was caused by elevated uric acid or changes of gut micaobiota. Besides, although we demonstrate the key bacteria that related with the progress of hyperuricemia, while the roles and mechanism of the bacteria in progress of hyperuricemia are unknown. Future work need focus on the mechanistic origins for intestinal barrier dysfunction in hyperuricemia. Furthermore, treatment of hyperuricemia by supplement or depletion of the key bacteria is also needed to be examined. The insights gained from our study hold the potential to enhance our mechanistic understanding and serve as a foundation for the identification of bacteria associated with uric acid levels and intestinal barrier function.

## Data availability statement

The datasets presented in this study can be found in online repositories. The names of the repository/repositories and accession number(s) can be found at: https://www.ncbi.nlm.nih.gov/, PRJNA982419.

## Ethics statement

The animal study was approved by Animal Research Ethics Committee of the Affiliated Hospital of Qingdao University. The study was conducted in accordance with the local legislation and institutional requirements.

## Author contributions

QL: Data curation, Formal analysis, Funding acquisition, Methodology, Writing – original draft. JZ: Methodology, Writing – original draft. CW: Methodology, Writing – review & editing. XY: Methodology, Writing – review & editing. YH: Formal analysis, Data curation, Supervision, Writing – review & editing. QZ: Formal analysis, Writing - review & editing. RY: Writing - review & editing, Methodology, Conceptualization. AS: Writing – review & editing, Supervision.
