# Corrigendum: A dynamics association study of gut barrier and microbiota in hyperuricemia

**DOI:** 10.3389/fmicb.2024.1358594

**Published:** 2024-02-12

**Authors:** Qiulan Lv, Jun Zhou, Changyao Wang, Xiaomin Yang, Yafei Han, Quan Zhou, Ruyong Yao, Aihua Sui

**Affiliations:** ^1^Medical Research Center, The Affiliated Hospital of Qingdao University, Qingdao, China; ^2^Laboratory Medicine, The Affiliated Hospital of Qingdao University, Qingdao, China

**Keywords:** hyperuricemia, gut microbiota, intestinal barrier, dynamic changes, dyslipidemia

In the published article, there was an error in Figure 2 as published. The *y*-axis in Figure 2A was incorrectly labelled as “Intestinal permeability FITC-dextran (ng/ml).” The correct label is “Intestinal permeability FITC-dextran (μg/ml).” Also, the *x*-axis in Figure 2B was incorrectly labelled as “FITC-dextran (ng/ml).” The correct label is “FITC-dextran (μg/ml).” The corrected Figure 2 and its caption appear below.

**Figure 2:**
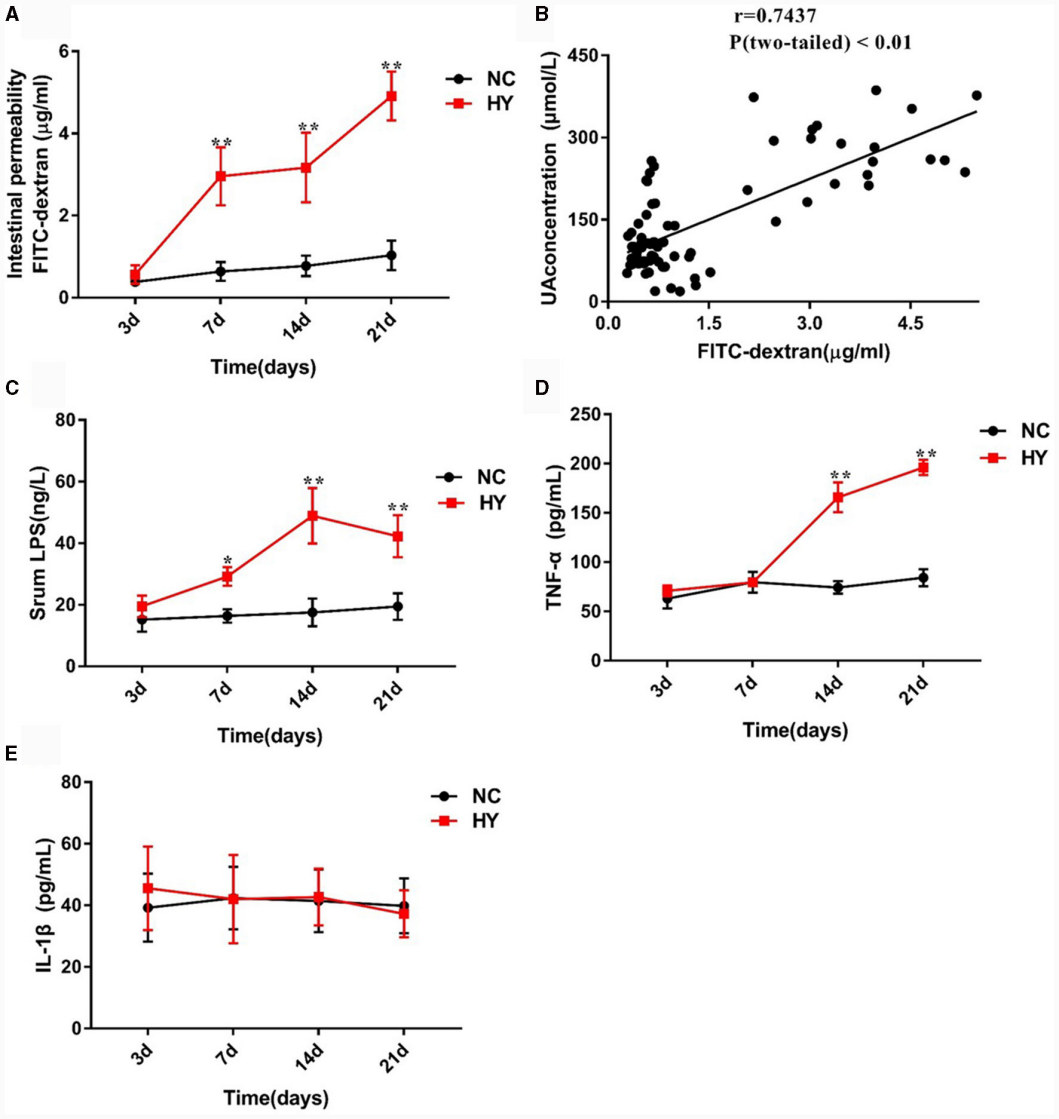
The dynamics of intestinal permeability and proinflammatory cytokines. **(A)** Intestinal permeability was detected in NC and HY mice at 3, 7, 14 and 21 days by administration of FD4, and levels of serum FITC-dextran present intestinal permeability. **(B)** Correlation of intestinal permeability and the uric acid was analyzed using Pearson correlation coefficients. **(C–E)** Serum LPS **(C)**, TNF-α **(D)** and IL-1β **(E)** levels were detected at 3, 7, 14, and 21 days using ELISA. *n* = 5 per group. Data are presented as the mean ± SEM. Data with different superscript letters are significantly different (*p* < 0.05) using one-way ANOVA followed by Tukey's multiple comparison post-test; *p < 0.05 and **p < 0.001 versus NC.

The authors apologize for this error and state that this does not change the scientific conclusions of the article in any way. The original article has been updated.

